# Polyamine Ligand-Mediated Self-Assembly of Gold and Silver Nanoparticles into Chainlike Structures in Aqueous Solution: Towards New Nanostructured Chemosensors

**DOI:** 10.1002/open.201300036

**Published:** 2013-10-31

## Abstract

Featured on this month′s cover are the results of collaborative work between the research group at the University Nova of Lisbon, Faculty of Science and Technology, BIOSCOPE-NanOmics in Portugal, and the group at the University of Santiago de Compostela, Faculty of Chemistry in Spain. The cover picture represents the ability of silver and gold nanoparticles to form 1D nanostructured materials in the presence of polyamine ligands. These self-assembled nanoparticles are able to sense Hg^2+^ metal ions in water and can potentially be used as chromogenic and fluorogenic sensors. For more details, see the Full Paper on page 200 ff.


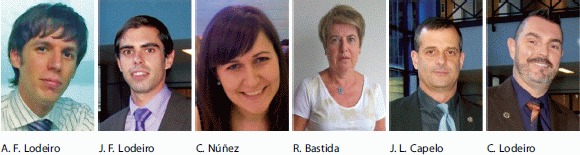



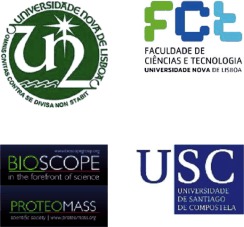


## How did the collaboration on this project start?

This collaboration started nearly 15 years ago with the purpose to use the expertise in the synthesis of polyamine acyclic and macrocyclic ligands and derivatives of the group at Santiago de Compostela, and the skills in nanochemistry and photophysical studies of the BIOSCOPE group for the development of new fluorescent nanostructured chemosensors.

## What is the most significant result of this study?

One significant result is the (surprising) ability of polyamine ligands bearing different donor atoms to interact as linkers with the surface of noble metal nanoparticles, thus, forming stable 1D nanostructured chains, and their use for the detection of metal ions, namely Hg^2+^, in solution with a significant enhancement in color and emission intensity.


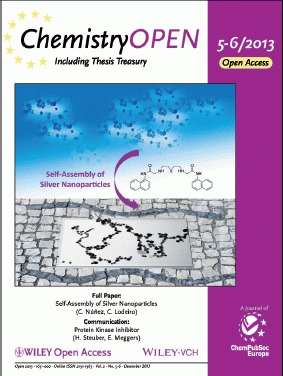


## What prompted you to investigate this topic/problem?

During the last ten years, our group has been involved in the synthesis, development and application of new, efficient and selective chemosensors for monitoring toxic and/or essential metal ions and anions in biological and environmental samples.

## What other topics are you working on at the moment?

Our groups are interested in the development of new emissive and nanostructured materials for application as fluorescent/colorimetric selective chemosensors and biomarkers, attached in nanoparticles, quantum dots, polymers and nanomaterials. Moreover, the group has strong experience in analytical proteomic studies applied to nano-biomedicine.

